# An Experimental Study on Properties of Pre-Coated Aggregates Grouting Asphalt Concrete for Bridge Deck Pavement

**DOI:** 10.3390/ma14185323

**Published:** 2021-09-15

**Authors:** Zhicheng Xiao, Wenke Huang, Kuanghuai Wu, Guihai Nie, Hafiz Muhammad Zahid Hassan, Bei Hu

**Affiliations:** School of Civil Engineering, Guangzhou University, Guangzhou 510006, China; xzc297055783@163.com (Z.X.); ngh706686573@163.com (G.N.); zahid.hassan197@gmail.com (H.M.Z.H.); 13026335960@163.com (B.H.)

**Keywords:** pre-coated aggregates, grouting asphalt concrete, deck pavement, high-temperature stability

## Abstract

Epoxy asphalt concrete, mortar asphalt concrete and Gussasphalt concrete are commonly used types of deck pavement materials in bridge deck pavement engineering. However, achieving the high-temperature stability and anti-fatigue performance of the deck pavement materials is still challenging. In order to reduce the rutting and cracking risks of the asphalt mixture, this paper proposed pre-coated aggregates grouting asphalt concrete (PGAC) for bridge deck pavement. Laboratory tests were conducted to determine the optimum grouting materials and to evaluate the mechanical performances of the PGAC material. Test results showed that the mechanical properties for PGAC with grouting material of high-viscosity-modified asphalt binder blending with mineral filler were superior to that of GMA-10 used for the Hong Kong-Zhuhai-Macau Bridge deck pavement. Microstructural analysis showed that the PGAC had a more stable skeleton structure compared to other typical aggregate mixtures. This study highlights the performances of the proposed PGAC and sheds light on the deck pavement material improvement of both high-temperature stability and anti-fatigue performance that could be achieved.

## 1. Introduction

Bridge deck pavement materials play a critical role in improving the driving comfort and structural durability of the bridge [1,2]. Epoxy asphalt concrete, mortar asphalt concrete and Gussasphalt concrete as three types of deck pavement materials have been widely used across the world [3,4,5].

As the surface material of a long-span bridge, Gussasphalt concrete is widely used in bridge deck pavement due to its unique water stability, fatigue resistance and anti-aging properties [6,7]. Many researchers have performed a lot of investigations to improve the performance of Gussasphalt concrete. Liu et al. [8] studied the performance of composite-modified asphalt with Trinidad Lake Asphalt (TLA) as a waterproof material for bridge deck pavement through the shear test, pull-out test and water permeability test. Chen et al. [9] put forward a three-stage characterization method of the Gussasphalt mixture. The primary characterization starts from evaluating the viscoelasticity of the binder, expands to the rheological behavior of asphalt-mineral filler cement, and finally, studies the engineering performance of the Gussasphalt mixture. Yang et al. [10] studied the influence of warm-mix modifiers RH and Sasobit on the road performance of the Gussasphalt mixture and concluded that warm-mix modifiers can effectively improve the fluidity of the Gussasphalt mixture but have adverse effects on the low-temperature crack resistance of the mixture.

With the increase of heavy traffic, the improvement of high-temperature stability and anti-fatigue performance has become an urgent problem to be solved in bridge deck pavement [11]. Although Gussasphalt mixture technology has been successfully applied globally, and a series of achievements have been made, Gussasphalt concrete containing a large portion of fine aggregates (aggregate size less than 2.36 mm) and a high content of asphalt binder is a typically suspended density mixture [12]. In this material, the small amount of coarse aggregates cannot form a stable skeleton [13]. Therefore, the structural strength of Gussasphalt concrete is greatly affected by the properties of the asphalt binder, which results in poor stability at high temperature and other distresses [14]. In order to form a stable mixture of skeleton structure and improve the high-temperature stability of the pouring asphalt mixture, a base penetration asphalt is added, such as Trinidad Lake Asphalt (TLA) [15,16].

Inspired by the concept of the skeleton density asphalt mixture, as commonly used in road pavement construction, a new type of Gussasphalt concrete, namely pre-coated aggregates grouting asphalt concrete (PGAC) for bridge deck asphalt pavement, is proposed in this study. The purpose of this study is to develop a new kind of asphalt mixture with high-temperature stability and excellent anti-fatigue performance, which can effectively reduce rutting and cracks of the surface layers on flexible bridges.

This novel method does not mix all the material components directly as in hot asphalt mixtures or Gussasphalt concrete. Instead, the coarse aggregate is firstly heated to a predetermined temperature and pre-coated with a specific content of asphalt binder. Then, the pre-coated aggregates are mixed and slightly compacted to form pre-mixed gravel with a stable skeleton. Finally, a hot asphalt binder or mastic is poured on the surface of the prepared gravel and then quickly flows into the mixture and fills the gravel gap. In this study, the gradation of aggregates was designed by the method of gradation parameter, and the asphalt dosage was determined by the idea of volume filling. The filling temperature and viscosity of six types of grouting materials were determined by a rotary viscosity experiment. At the same time, the high-temperature stability and low-temperature crack resistance of PGAC and GMA-10 were compared by the rut test and the three-point bending test. The construction layer thickness of PGAC was determined by the pull-out experiment. Finally, the digital image processing technology was used to analyze the contact characteristics and average inclination angle of coarse aggregate, and the analysis results show that PGAC has a better ability to resist deformation compared with other mixtures. The purpose of this study is to develop a new kind of skeleton Gussasphalt concrete for bridge deck asphalt pavement that has good high-temperature stability and low-temperature crack resistance properties.

## 2. Materials and Methods

### 2.1. Materials

Three types of asphalt binders, including styrene–butadiene–styrene (SBS)-modified asphalt binder (Shell Road Solutions Xinyue Co. Ltd. Foshan, Guangdong, China), high-viscosity (HV)-modified asphalt binder (Shell Road Solutions Xinyue Co. Ltd. Foshan, Guangdong, China) and super-high-viscosity (SHV)-modified asphalt binder (Shell Road Solutions Xinyue Co. Ltd. Foshan, Guangdong, China) were selected in this study. Crushed basalt stone was used as coarse and fine aggregates and the mineral filler was limestone powder. The detailed properties of asphalt binder, aggregate and mineral filler are provided in Table 1 and Table 2, respectively.

### 2.2. Aggregate Gradation

To maximize the density in the PGAC, the design of aggregate gradation was performed with the multilevel mixing method initially proposed by Lees et al. [17]. The aggregate gradation is designed through several stages of blending of coarse and fine aggregates to obtain the optimum ratio of mineral aggregates by maximizing mixture density [18]. The maximum and minimum nominal aggregate size used in this study were 13.2 and 2.36 mm, respectively. Therefore, the aggregate gradation was designed with a multilevel mixing method to utilize 13.2–2.36 mm aggregates to form the skeleton in the mixture. A dry tamping test was applied to determine the optimal ratio of different sizes of aggregates by obtaining the density and void ratio of the aggregate mixture.

**Figure 1:**
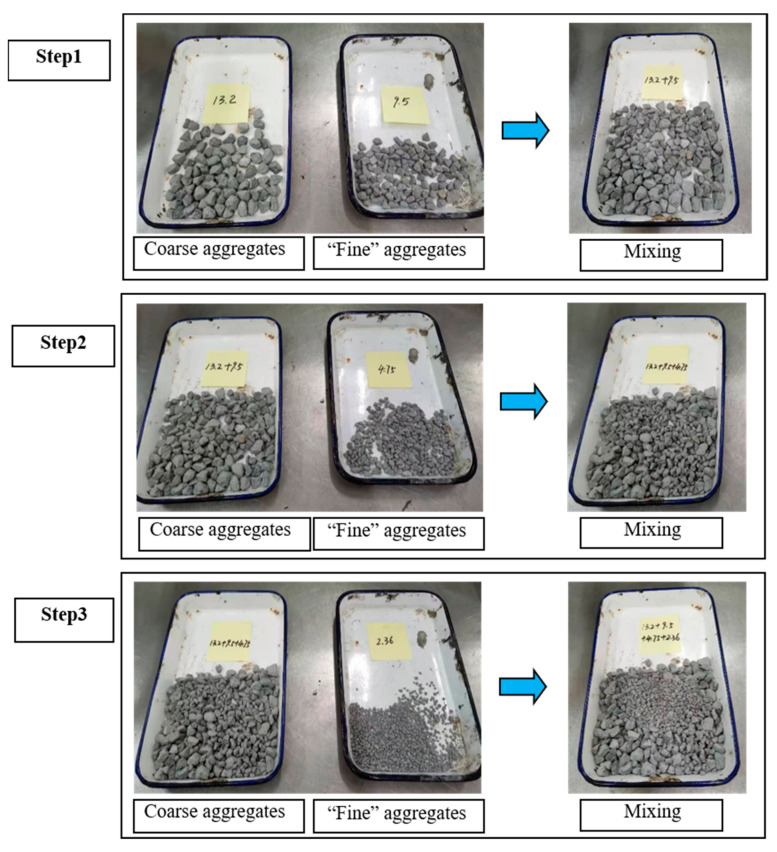
Flow chart of the multilevel mixing method.

In this study, the aggregate gradations were carried out in three steps. Figure 1 shows the gradation designed procedure of the multilevel mixing method. The first step was to mix the “coarse aggregate” (13.2 mm) with “fine aggregates” (9.5 mm), and then the dry tamping test was used to determine the optimum ratio of “coarse aggregate” and “fine aggregate”. The second step was to blend the ‘‘coarse aggregate” (13.2 and 9.5 mm) taken from the first step with “fine aggregates” (4.75 mm), and the optimum ratio of “coarse aggregate” and “fine aggregate” was determined by the dry tamping test. The third step involved mixing the “coarse aggregate” (13.2, 9.5 and 4.75 mm) with “fine aggregates” (2.36 mm) and determining the optimum proportion of aggregates through a dry tamping test. The aggregate gradation of the PGAC used in this study can be seen in Table 3.

To determine the optimum grouting materials for the PGAC, a total of six types of asphalt concrete, namely A, B, C, D, E and F, shown in Table 3, were designed. The differences in these PGACs involved the types of asphalt binders and the contents of mineral filler. The primary role of the asphalt or asphalt mastic in the PGAC is to fill the voids of the skeleton formed by the aggregates. The asphalt content can be calculated with Equation (1), expressed as:

(1) $$Q=VCA_{DRC}\times \frac{\rho_{a}}{\rho_{sc}\times \left(1+r\times \frac{\rho_{a}}{\rho_{p}}\right)}$$

where *Q* is the asphalt content,$\;VCA_{DRC}$ is the void ratio of coarse aggregate in the dry tamping test, $\rho_{sc}$, $\rho_{a}$, $\rho_{p}$ are dry tamping density of aggregates, density of asphalt binder and density of mineral filler respectively, and *r* is the filler-to-asphalt ratio. Contents of asphalt binder in the six types of asphalt concrete, namely A, B, C, D, E and F, were 21.8%, 15.3%, 22.0%, 15.8%, 22.6% and 16.6%, respectively.

### 2.3. Grouting Scheme

**Figure 2:**
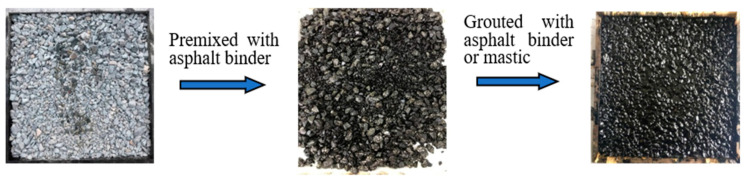
Flow chart of the grouting scheme.

In the laboratory experiment, the graded aggregates and the asphalt binder (or asphalt mastic) were heated to a given temperature in the oven. Then, the asphalt binder with 0.6% content was added into the graded aggregates and mixed evenly for 90 s. The graded aggregates pre-mixed with asphalt binder were placed in the trial tank and then slightly compacted and scraped flat. After that, the asphalt binder (or asphalt mastic) with the best content calculated with Equation (1) was grouted into the mixture. Figure 2 shows the procedure of the grouting program.

## 3. Results

### 3.1. Optimum Grouting Materials for Pre-Coated Aggregates Grouting Asphalt Concrete

In this study, six types of grouting material, including SBS-modified asphalt binder, SBS-modified asphalt binder blended with mineral filler, HV-modified asphalt binder, HV-modified asphalt binder blended with mineral filler, SHV-modified asphalt binder and SHV-modified asphalt binder blended with mineral filler, were selected.

#### 3.1.1. Viscosity–Temperature Tests

In order to ensure that the grouting materials can fill the voids of the skeleton formed by the aggregates, the grouting materials must have sufficient fluidity. Kinematic viscosity is one of the rational indicators to characterize the workability of asphalt during the construction stage. According to the Standard Test Methods of Bitumen and Bituminous Mixtures for Highway Engineering in China (JTG E20-2011, T0625), the recommended kinematic viscosity of asphalt binder for construction is 0.28 Pa·s. In this case, the actual construction temperature of the grouting materials can be determined by the viscosity–temperature curves.

**Figure 3:**
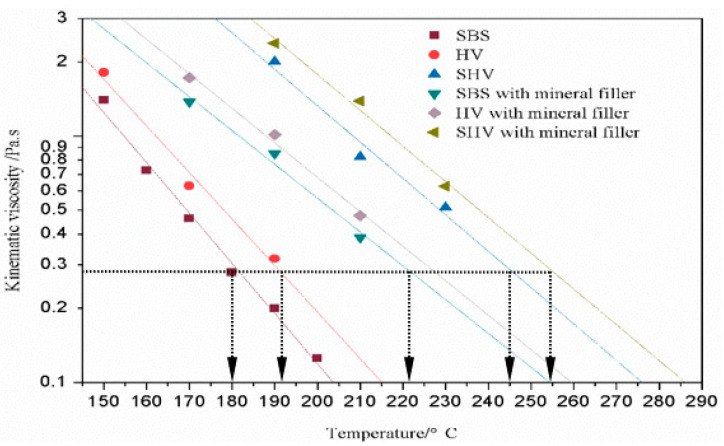
Viscosity–temperature curves of six types of grouting material.

A No. 27 rotor was used to determine the kinematic viscosity of the six grouting materials at a rate of 20 r/min. The viscosity–temperature curves of the six grouting materials are shown in Figure 3. We found that the viscosity of the six types of asphalt binders or asphalt mastic decrease linearly with the increasing temperature. Temperatures corresponding to the 0.28 Pa·s viscosity of SBS-modified asphalt binder, SBS-modified asphalt binder blended with mineral filler, HV-modified asphalt binder, HV-modified asphalt binder blended with mineral filler, SHV-modified asphalt binder and SHV-modified asphalt binder blended with mineral filler were 180, 223, 192, 227, 245 and 255 °C, respectively.

#### 3.1.2. Rutting Tests

**Figure 4:**
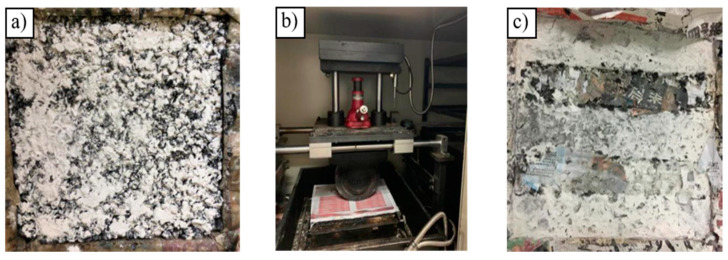
Rutting test of the PGAC: (**a**) spreading mineral filler on surface of the specimen, (**b**) rutting tester, (**c**) top view of the specimen after testing.

High-temperature stability was selected for determining the optimum grouting media for PGAC from the six types of grouting materials. Rutting tests of PGAC were conducted at a temperature of 60 °C with a wheel pressure of 0.7 MPa according to the Standard Test Methods of Bitumen and Bituminous Mixtures for Highway Engineering in China (JTG E20-2011, T0719). To eliminate the adhesion between the specimen and the wheel of the rutting tester, a layer of mineral filler can be spread on the surface of the asphalt specimen and then a piece of newspaper is placed over it before testing. Figure 4 shows the rutting test procedure of the PGAC.

**Figure 5:**
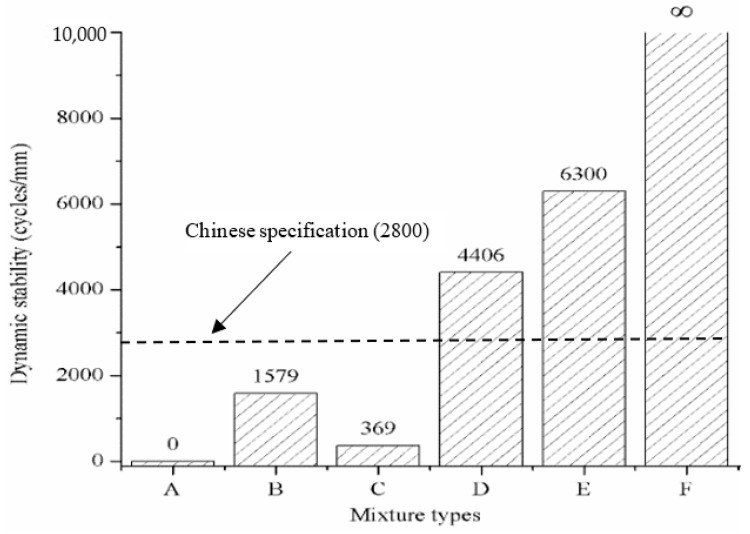
Dynamic stability of six types of PGAC.

The dynamic stability of the six types of PGAC, namely A, B, C, D, E and F, is illustrated in Figure 5. The rutting depth of Type-A PGAC exceeded the range of the displacement sensor of the rutting tester. Therefore, the dynamic stability can be considered zero, which means that the SBS-modified asphalt binder is not suitable as a grouting material in the PGAC domain. The reason is that the SBS-modified asphalt binder cannot stabilize the skeleton of the graded aggregates at high temperature due to its relatively lower dynamic viscosity. For graded aggregates grouted with SBS-modified asphalt binder blended with mineral filler and HV-modified asphalt binder, the dynamic stabilities are 1579 and 369 cycles/mm, respectively. However, the dynamic stabilities are less than the recommended value of 2800 cycles/mm for a modified asphalt mixture corresponding to the Technical Specifications for Construction of Highway Asphalt Pavements in China (JTG F40-2004). With regard to Type-D and Type-E PGAC, the dynamic stabilities increased sharply, reaching 4406 and 6300 cycles/mm, respectively. The dynamic stability tends to be infinity for graded aggregates grouted with SHV-modified asphalt binder blended with mineral filler. It can be concluded from Figure 5 that graded aggregates grouted with higher viscosity media seem to have greater dynamic stability values.

Considering the energy consumption and cost of materials, the grouting material of HV-modified asphalt binder blended with mineral filler (Type-D, referring to Table 3) was selected as the optimum grouting media, and the properties of PGAC based on this grouting media in the following sections were evaluated.

### 3.2. Mechanical Performance of PGAC

In this section, the mechanical performance of PGAC was compared with that of gussmortar asphalt (GMA-10) used for the Hong Kong-Zhuhai-Macau Bridge deck pavement. Details of the GMA-10 experimental results can be found in [19].

#### 3.2.1. High-Temperature Stability

The rutting test of GMA-10 used for the Hong Kong-Zhuhai-Macau Bridge deck pavement has the same test setup compared to that of PGAC selected in this study. The dynamic stability of GMA-10 recommended for the Hong Kong-Zhuhai-Macau Bridge deck pavement was from 300 to 700 cycles/mm at a temperature of 60 ℃. According to Figure 5, the dynamic stability for grouting material of HV-modified asphalt binder blended with mineral filler adapted for mechanical performance analysis in this section reached 4406 cycles/mm, which is significantly higher than that of GMA-10. This is primarily because of the high contents of asphalt binder and fine aggregates in the GMA-10, making the coarse aggregates remain in suspension state and resulting in weakening the rutting deformation resistance, while the PGAC forms a stable skeleton when the voids of the skeleton formed by the aggregates are filled with grouting media. The experimental results show that the Type-D PGAC selected in this study has a better rutting resistance performance than that of GMA-10.

#### 3.2.2. Low-Temperature Crack Resistance

**Figure 6:**
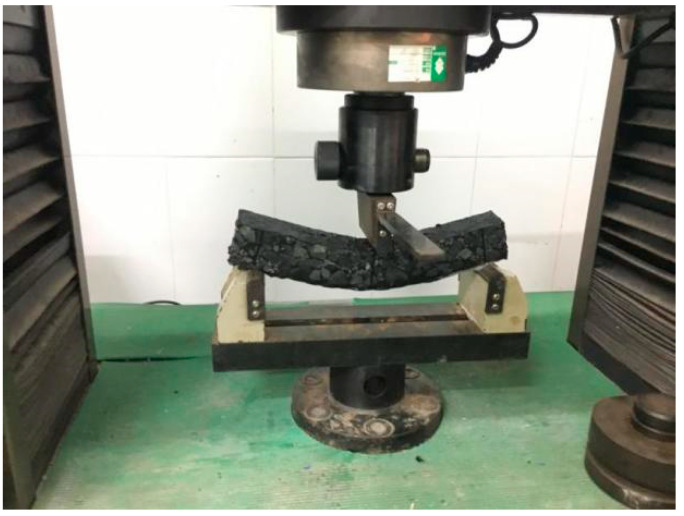
Three-point bending test.

Three-point bending tests were conducted to evaluate the low-temperature crack resistance of PGAC and GMA-10 according to the Standard Test Methods of Bitumen and Bituminous Mixtures for Highway Engineering in China (JTG E20-2011, T0715). The testing specimens with the dimensions of 250 mm × 30 mm × 35 mm in length, width and height were cut from prepared slab specimens. The temperature and loading rate of the three-point bending tests were selected as −10 ℃ and 50 mm/min, respectively. The test setup is illustrated in Figure 6. Specifically, testing results including the flexural tensile strength, stiffness modulus and maximum flexural strain were used to characterize the low-temperature crack resistance, which can be calculated with Equations (2)–(4), expressed as:

(2) $$R_{B}=\frac{3\times L\times P_{B}}{2\times b\times h^{2}}\;$$

(3) $$\varepsilon_{B}=\frac{6\times h\times d}{L^{2}}\;$$

(4) $$S_{B}=\frac{R_{B}}{\varepsilon_{B}}\;$$

where, *L* is the span length of the specimen (mm), *h* is the mid-span height of the specimen (mm), *b* is the mid-span width of the specimen (mm), *d* is the mid-span deflection of the specimen at failure (mm), *P_B_* is the maximum load at failure (N), *R_B_* is the flexural tensile strength (MPa), *ε_B_* is the maximum flexural strain and *S_B_* is the stiffness modulus (PMa).

Generally, an asphalt mixture with higher flexural tensile strength tends to have stronger resistance to failure at low temperatures. Similarly, an asphalt mixture with a larger maximum flexural strain has a better ability to withstand the deformation at low temperatures. In other words, an asphalt mixture has the best low-temperature crack resistance property under the ideal condition of high flexural tensile strength and large maximum flexural strain. Therefore, the flexural tensile strength and maximum flexural strain of asphalt mixture are the two most important indexes to evaluate its low-temperature crack resistance ability.

As shown in Table 4, the flexural tensile strength of PGAC is very close to that of GMA-10, indicating that PGAC has the same ability to resist damage as GMA-10. The maximum flexural strain of PGAC reaches 7502 micro-strains, which is much larger than that of GMA-10. The results of the experiment show that the PGAC has the same low-temperature crack resistance as GMA-10 but possesses better flexibility than GMA-10.

#### 3.2.3. Interfacial Bond Property

**Figure 7:**
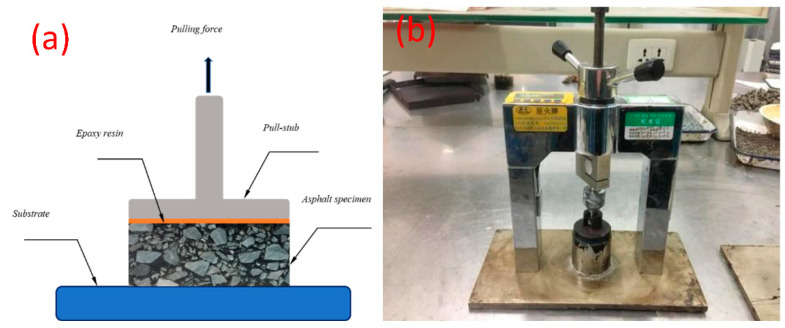
Pull-out system for PGAC: (**a**) cross-section view of the pull-out test and (**b**) pull-out testing.

In order to better evaluate the bonding performance of PGAC to the base, and verify whether PGAC has improved compared with the traditional asphalt penetration pavement material, in this section, the pull-out tests were conducted to compare the interface strength of PGAC with that of GMA-10 on steel and cement concrete substrates. Three types of PGAC cylinder specimens with a diameter of 50 mm and heights of 40, 60 and 80 mm were formed in the thin-walled stainless-steel pipes on steel and cement concrete substrates. After being cured for 24 h, the specimens were bonded with the pull-stub by epoxy resin, as shown in Figure 7.

**Figure 8:**
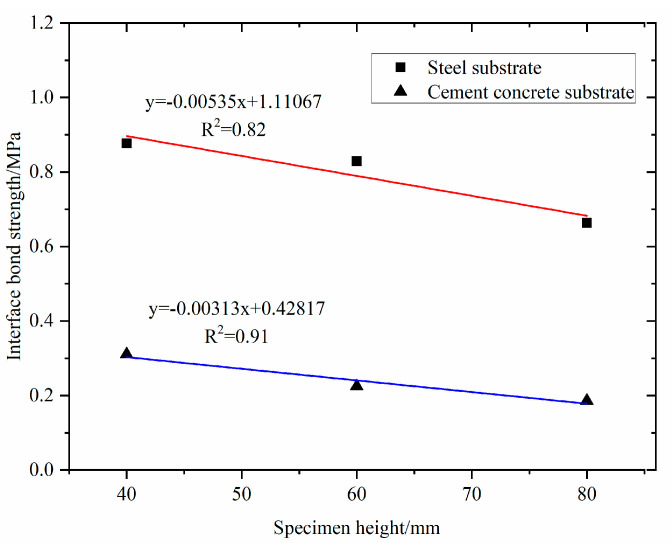
Interfacial bond strength of PGAC with different heights on different substrates.

**Figure 9:**
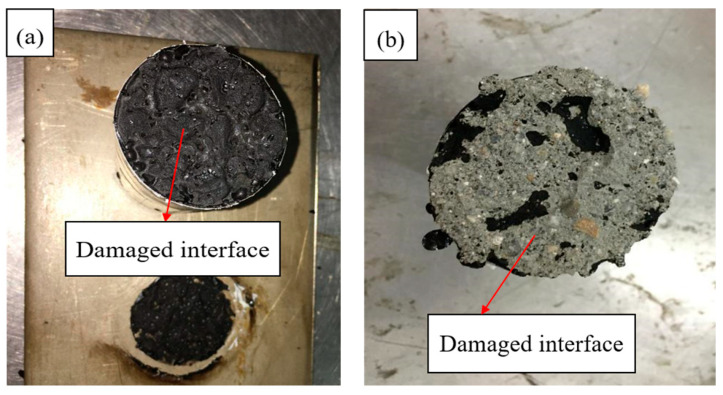
Damaged interface: (**a**) damaged interface of PGAC specimen on steel substrate, (**b**) damaged interface of PGAC specimen on cement concrete substrate.

The pull-out tests of the PGAC specimens were conducted at temperature 25 ℃. Interfacial bond strength of PGAC with different heights on two different substrates are shown in Figure 8. It can be seen from Figure 8 that the interface bond strength of PGAC on the steel substrate is higher than that on the cement concrete substrate. The reason why this happens is because the damaged interface of PGAC on the steel substrate is located at the bottom of the PGAC specimen, as shown in Figure 9a, while the damaged interface of PGAC on the cement concrete substrate mainly occurs on the cement concrete, as shown in Figure 9b. The phenomenon shows that the interface bond strength of PGAC on the cement concrete substrate greatly decreases due to the damage of the cement concrete base itself.

It can also be concluded from Figure 8 that the interfacial bond strength of PGAC decreases with the increase of the specimen thickness, primarily because the temperature of the asphalt mastic gradually decreased in the process of grouting, and the viscosity of the asphalt mastic gradually increased. When the viscosity of the asphalt mastic increased to a certain extent, the fluidity of the asphalt mastic decreased, and the bottom of the specimen cannot be completely filled with asphalt mastic. Therefore, when the height of the PGAC specimen is small to a certain extent, the asphalt mastic can easily reach the bottom of the specimen and fully contact the substrate.

**Figure 10:**
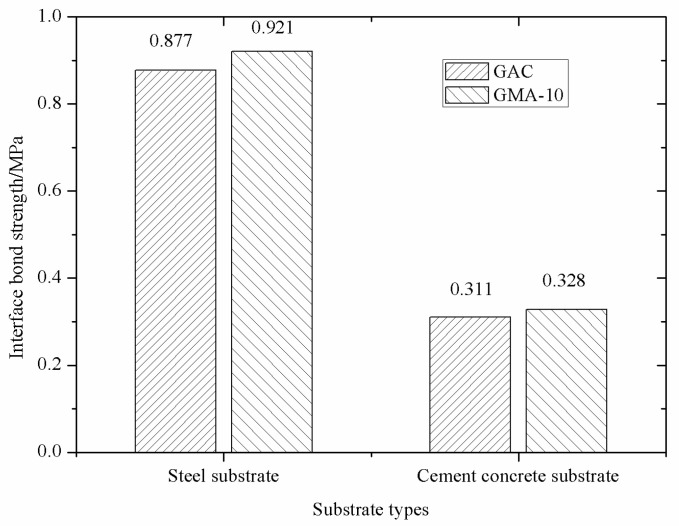
Interfacial bond strength of PGAC and GMA-10 on different substrates.

The interfacial bond strengths of PGAC with a layer thickness of 4 cm on steel and cement concrete substrates were compared with that of GMA-10, as shown in Figure 10. It can be seen from Figure 10 that the interface bond strengths of PGAC on two different substrates are equivalent to that of GMA-10. Therefore, it is suggested in this study that the construction layer thickness of this PGAC should be 4~6 cm. When the construction layer thickness exceeds to 8 cm, it is suggested to coat the waterproof material on the substrate before paving the PGAC.

### 3.3. Microstructural Analysis

**Figure 11:**
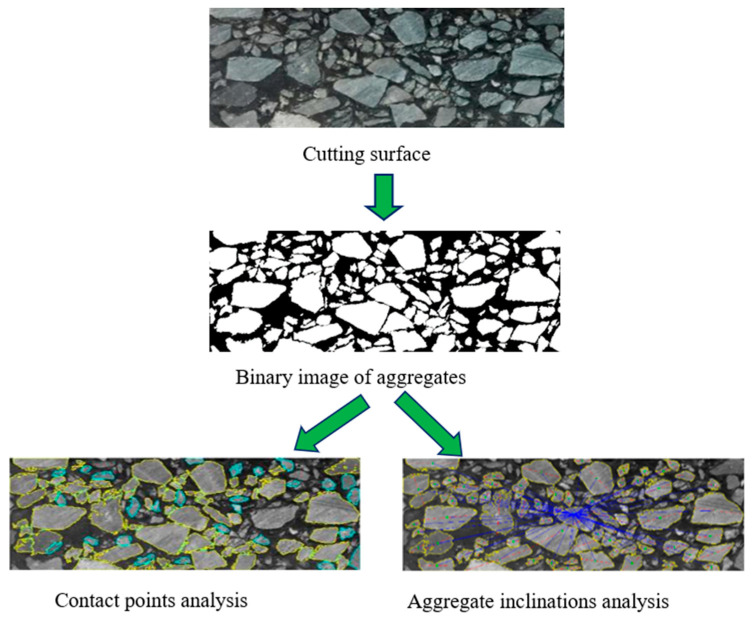
Procedures of microstructural analysis.

**Figure 12:**
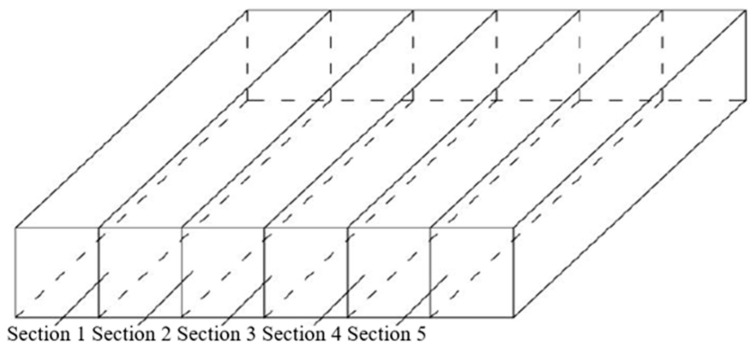
Schematic diagram of cutting.

In order to analyze the distribution of internal structural particles of PGAC more intuitively, digital image processing technology was applied to study the contact characteristics and mean inclination of coarse aggregates. The procedures of microstructural analysis of the coarse aggregates can be seen in Figure 11. The first step is to use a cutting machine to cut the asphalt mixture slab into 6 pieces to obtain 5 groups of interfaces (each group has 2 identical sections, a total of 10 sections). In this way, the internal cross-section image of the specimen can be obtained. The cutting diagram is shown in Figure 12. Then, digital image processing technology was utilized to acquire a binary image. Particles that are connected to each other is a common phenomenon in the segmentation process of the binary image, which increases the difficulty of microstructural analysis. An improved morphological multiscale algorithm based on several different sizes of structural elements was introduced to segment the coarse aggregate adhesion images to overcome this difficulty. Details of this segmentation algorithm can be found in our previous research [20]. After that, contact points and inclinations were analyzed based on the pretreated binary image. Details of the calculation of contact points and inclinations can be found in our previous research [21]. The number of contact points and inclinations of the cross-section of PGAC were studied compared with AC-13, SMA-13, SUPERPAVE-13 and OGFC-13 asphalt mixtures. Contact points and inclinations of AC-13, SMA-13, SUPERPAVE-13 and OGFC-13 asphalt mixtures have been analyzed in [22].

#### 3.3.1. Contact Point Analysis

**Figure 13:**
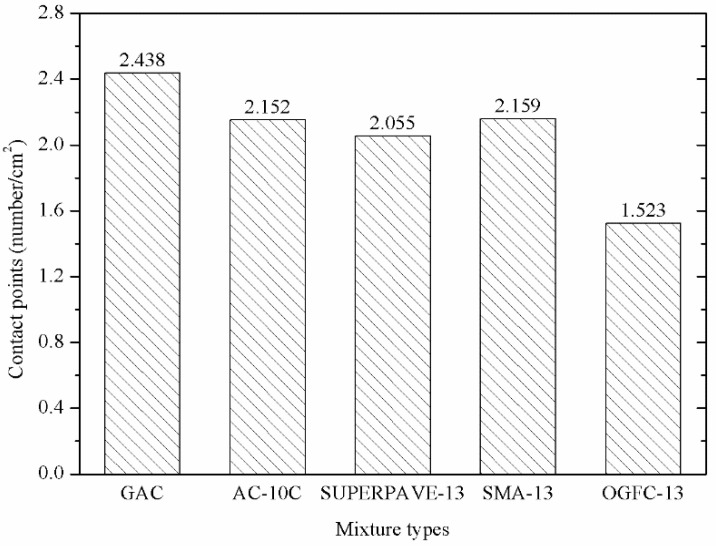
Comparison of contact points of PGAC with four other kinds of asphalt mixture.

The internal coarse aggregates of asphalt mixture formed a skeleton by friction and interlocking to bear the external load. To a certain extent, the density of aggregate contact points reflects whether the aggregates are fully contacted as well as the rutting resistance properties of the asphalt mixture. In this study, the numbers of contact points (unit: number/cm^2^) were used as the characterization parameter of contact point density. A comparison of contact points for PGAC with four other kinds of asphalt mixture can be seen in Figure 13.

According to Figure 13, the number of contact points of the cross-section for PGAC reaching 2.438 cm^2^ is the largest, which means it is the maximum density of PGAC. This occurs because the aggregate of PGAC does not contain fine aggregates whose particle sizes are less than 2.36 mm, which ensures that fine aggregates will not separate the skeleton of coarse aggregates during the construction process and the coarse aggregates are kept in the crammed state. Therefore, the contact characteristic of the internal structure of PGAC is relatively better than that of the other four dense skeleton types of asphalt mixtures, which is beneficial to the rutting resistance of the mixture. In other words, the PGAC can maintain good high-temperature stability with a high asphalt binder content.

#### 3.3.2. Inclination Analysis

**Figure 14:**
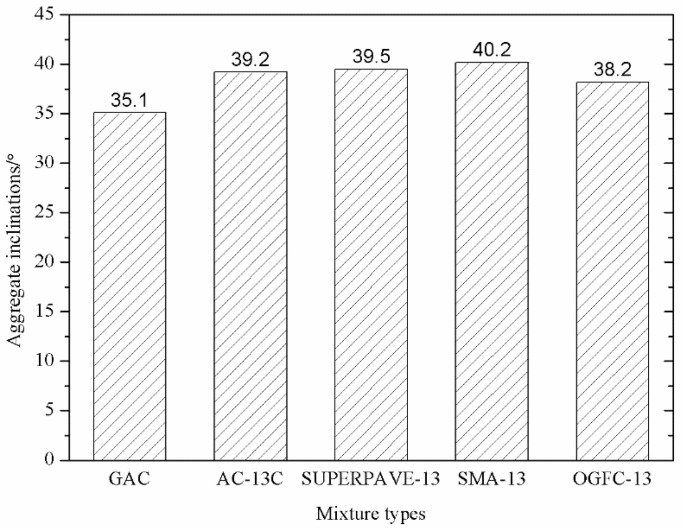
Comparison of inclination of coarse aggregates of five types of asphalt mixtures.

The inclination of coarse aggregates is also an important indicator that reflects the degree of stabilization of the internal structure of the asphalt mixture. An asphalt mixture with a minor degree of inclination can be more stable. Figure 14 shows the comparison between the inclination of coarse aggregates in PGAC and that in the other four typical mixed mixtures.

As shown in Figure 14, the inclination of coarse aggregates of the five types of asphalt mixtures is in the following order: PGAC < OGFC-13 < AC-13 < SUPERPAVE-13 < SMA-13. The inclination of coarse aggregates for PGAC is the smallest, which indicates that PGAC has the most stable skeleton structure compared with the four other mixes. This finding is also consistent with the conclusion in Section 3.3.1.

## 4. Conclusions

In this paper, PGAC for bridge deck pavement was proposed to improve the high-temperature stability and low-temperature cracking resistance properties of an asphalt mixture. Laboratory tests were conducted to determine the optimum grouting materials and to evaluate the mechanical performances of the PGAC material. Then, digital image processing technology was applied to study the contact characteristics and mean inclination of coarse aggregates to explain why the proposed PGAC can maintain stability at high temperatures with a high content of asphalt binder. The following conclusions can be drawn:

(1) The actual construction temperature of each binder was determined by the viscosity–temperature curve. By comparing the rutting resistance performance of PGAC prepared by six grouting materials, and considering the factors of dynamic stability and energy consumption, the Type-D grouting material (HV-modified asphalt binder mixed with mineral filler) was selected as the best grouting medium.
(2) The dynamic stability for PGAC with Type-D grouting material reached 4406, which was significantly higher than that of GMA-10 used for the Hong Kong-Zhuhai-Macau Bridge deck pavement. The flexural tensile strength of PGAC was very close to that of GMA-10, but the maximum flexural strain of PGAC reached 7502 micro-strain, largely greater than that of GMA-10, which indicates that the PGAC had the same low-temperature crack resistance as GMA-10 but possesses better flexibility than GMA-10. Four-point bending experiments will be conducted in the subsequent research to verify the anti-fatigue performance of GAC against cyclic stress.
(3) The interface bond strengths of PGAC on steel and cement concrete substrates were equivalent to those of GMA-10. The structure layer thickness of PGAC was recommended to be 4–6 cm. It is suggested to coat the waterproof material on the substrate before paving PGAC when the construction layer thickness exceeds 8 cm. The number of contact points and mean inclination of coarse aggregates for PGAC were superior to other typical aggregate mixtures, which means that the PGAC had a more stable skeleton structure.

## Figures and Tables

**Table 1:** Measurements of asphalt binder.

Physical Properties	Asphalt Types		
	SBS	HV	SHV
Penetration at 25 °C (1/10 mm)	54.4	48.1	42.8
R&B softening point (°C)	88.6	93	102
Ductility at 5 °C (cm)	32	31.5	30.5
Kinematic viscosity at 190 °C (Pa·s)	0.2	0.32	2.0
Separation at 163 °C, 48 h (°C)	1.0	1.5	1.5

**Table 2:** Aggregate and mineral filler properties.

Materials	Physical Properties	Test Results
Coarse Aggregate	Relative apparent density	2.69
	Water absorption	0.20%
	Aggregate crushed value	20%
	Los Angeles abrasion value	26%
	Flakiness and elongation index	13%
Mineral Filler	Relative apparent density	2.40
	Moisture content	0.12%

**Table 3:** Gradations of asphalt mixture.

Sieve Size (mm)	Percentage Passing (%)					
	A	B	C	D	E	F
16	100	100	100	100	100	100
13.2	71.2	71.2	71.2	71.2	71.2	71.2
9.5	50.7	50.7	50.7	50.7	50.7	50.7
4.75	30.1	30.1	30.1	30.1	30.1	30.1
2.36	0	0	0	0	0	0
Asphalt binder	SBS	SBS	HV	HV	SHV	SHV
Mineral filler (%)	0	19.5	0	21.8	0	24.9
Asphalt content (%)	21.8	15.3	22.0	15.8	22.6	16.6

**Table 4:** Bending test results at a temperature of −10 ℃.

Mixture Type	Flexural Tensile Strength/MPa	Maximum Flexural Strain/με	Stiffness Modulus/MPa
PGAC	6.46	7502	861
GMA-10	6.94	3714	1867

## Data Availability

Not applicable.
